# Differential expression of peroxiredoxin 3 in laryngeal squamous cell carcinoma

**DOI:** 10.18632/oncotarget.13838

**Published:** 2016-12-09

**Authors:** Hua Zhang, Xuexia Liu, Lei Chen, Li Cai, Ning Li, Peng Zhu, Jian Chen, Xicheng Song, Guojun Li

**Affiliations:** ^1^ Department of Otolaryngology-Head and Neck Surgery, Yuhuangding Hospital of Qingdao University, Yantai, China; ^2^ Department of Head and Neck Surgery, The University of Texas MD Anderson Cancer Center, Houston, TX, USA; ^3^ Department of Central Laboratory, Yuhuangding Hospital of Qingdao University, Yantai, China; ^4^ Department of Clinical Laboratory, Yuhuangding Hospital of Qingdao University, Yantai, China; ^5^ Department of Pathology, Yuhuangding Hospital of Qingdao University, Yantai, China; ^6^ Department of Epidemiology, The University of Texas MD Anderson Cancer Center, Houston, TX, USA

**Keywords:** peroxiredoxin 3, laryngeal squamous cell carcinoma, Hep-2 cell line, oxidative stress

## Abstract

Peroxiredoxin (PRDX) proteins are involved in carcinogenesis. PRDX3, which is predominantly localized in mitochondria and up-regulated in several human cancers, seems to confer increased treatment resistance and aggressive phenotypes. This study examined the expression profile of PRDX3 and its possible clinical value in laryngeal squamous cell carcinoma (LSCC). The expression of PRDX3 in LSCC samples was confirmed by Western blotting and further analyzed by immunohistochemistry in LSCC samples of different clinical pathological stages. The results showed that up-regulated expression of PRDX3 was observed in LSCC and associated with poor differentiation (P < 0.01), primary tumor location, N category and tumor stage (P < 0.05). Knockdown of PRDX3 in the Hep-2 laryngeal carcinoma epithelial cell line significantly enhanced Hep-2 cells’ apoptosis and inhibited their proliferation and migration. Taken together, our results suggest that PRDX3 has substantial clinical impact on the progression of LSCC and shed new light on the role of PRDX3 in treatment resistance and aggressive phenotypes in LSCC.

## INTRODUCTION

Head and neck cancer (HNC) is one of the most malignant carcinomas in the world [1]; 25% of HNCs are laryngeal carcinomas [2, 3], and 95-98% of these are laryngeal squamous cell carcinoma (LSCC) [4]. According to American Cancer Society data, death from LSCC accounted for 0.7% of cancer-related deaths in 2009 and its incidence is increasing [5]. Although advances in surgical management, radiotherapy, and chemotherapy have been observed over the last 3 decades, clinical outcomes of LSCC have not significantly improved due to tumor recurrence and metastasis [6]. Despite an improved 5-year survival rate, 30-40% of laryngeal carcinoma patients still die of tumor metastasis [3].

In a previous study, we reported increased reactive oxygen species (ROS) levels in LSCC serum, implicating ROS in the development of LSCC [7]. ROS is well known to be involved in the development of cancers [8], and elevated levels of ROS promote cancer development by inducing genomic instability, modifying gene expression, and participating in signaling pathways. However, the nature of this association is both complex and at times paradoxical. A large number of studies have also shown that excessive ROS induce cell apoptosis or autophagy in cells, excessive ROS can cause cell necrosis. Chemotherapy and radiotherapy for cancer may be selectively toxic to tumor cells because they augment oxidant stress and push these already stressed cells beyond their limit [9–11].

The degree of oxidant stress in a cell reflects a balance between the rate of ROS production and the activity of scavenging systems that detoxify them. Organisms have developed multiple antioxidant systems to protect against oxidative damage. Peroxiredoxin (PRDX) is the main family of antioxidant proteins. The mammalian PRDXs--PRDX1, PRDX2, PRDX3, PRDX4, PRDX5, and PRDX6--have conserved reactive cysteine residues in their active sites by which hydrogen peroxide (H_2_O_2_) is reduced [12]. PRDXs play important roles in cancer development, progression, and recurrence. Recently, the involvement of ROS signaling in tumor metastasis was highlighted [13, 14], but the expression profiles of PRDXs in carcinogenesis have not been systematically investigated, especially in LSCC. In the present study, we investigated the expression patterns of PRDXs in LSCC to identify a useful target for the early diagnosis and treatment of LSCC.

## RESULTS

### Up-regulated PRDX3 in LSCC tumor tissues

**Figure 1:**
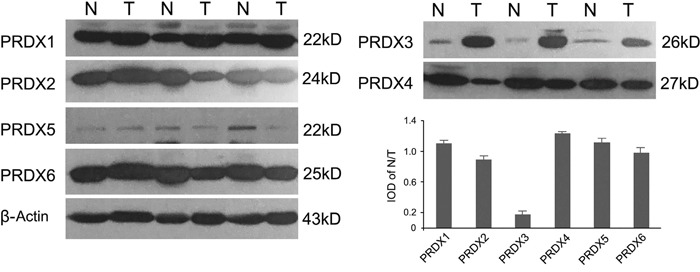
Representative expression profiles of PRDXs in LSCC tissues (n=48) Normal adjacent tissues and tumor tissues were analyzed by Western blotting and quantified by integrated optical density (IOD). N: Normal adjacent tissue; T: Tumor tissue. β-actin expression was used as an internal standard.

**Figure 2:**
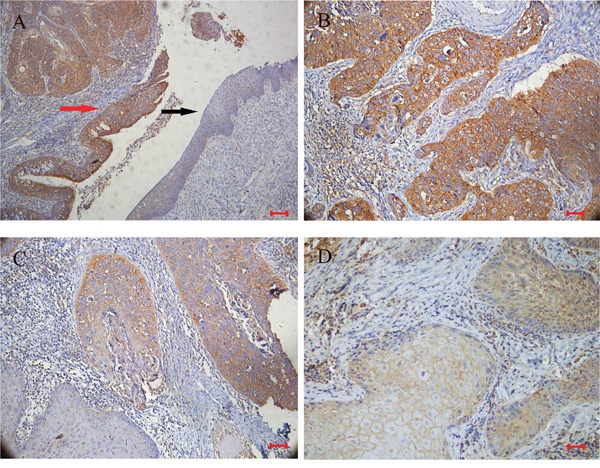
Representative immunohistochemical staining for PRDX3 in sections of LSCC (n=48) **A**. Positive staining in cancer tissues (red arrow), and negative staining in adjacent normal epithelium (black arrow) (×100; bar=100*μm*); **B**. strong immunostaining in poorly differentiated LSCC samples (×200; bar=50*μm*); **C**. moderate immunostaining in moderately differentiated LSCC samples (×200; bar=50*μm*); **D**. weak immunostaining in well-differentiated LSCC samples (×200; bar=50*μm*).

A protein profile of PRDXs in LSCC was obtained using Western blotting in tumor tissues and normal adjacent tissues from 48 cases of LSCC. As shown in Figure 1, PRDX3 was significantly up-regulated in tumor tissues of LSCC compared with normal adjacent tissues. Significant differences in expression were not observed for the other PRDXs. Subsequently, cellular localization of PRDX3 in LSCC tissues was detected by immunohistochemistry. PRDX3 was mainly expressed in the cytoplasm. Compared to the normal tissues, PRDX3 was dramatically up-regulated in the laryngeal tumor tissues (Figure 2A). The differentiated tissues had different staining intensity (Figure 2B, 2C, 2D). Strong immunostaining (Grade III) was observed in poorly differentiated LSCC samples, moderate immunostaining (Grade II) was observed in moderately differentiated LSCC samples, and weak immunostaining (Grade I) was observed in well-differentiated LSCC samples.

### Association of expression of PRDX3 with clinical characteristics and survival

To investigate the role of PRDX3 in the progression of LSCC, the association between multiple clinical features of LSCC patients and the expression of PRDX3 was analyzed. The clinical features of these patients are provided in Table 1. As shown in Table 2, twenty-three patients (47.9%) had Grade I, 33.3% (16 of 48) had Grade II, and 18.8% (9 of 48) had Grade III. Grade I expression of PRDX3 was also associated with glottic tumor location, lower N stage and tumor stage (all P<0.05), while no significant difference in PRDX3 expression was observed for sex, age, tobacco smoking, and T stage (P > 0.05).

**Table 1:** Characteristics of LSCC patients (n=48)

Variable	N (%)
No. of patients	48
Age	
Median age	65
Range	41-81
Sex	
Male	45(93.8)
Female	3(6.2)
Tobacco smoking	
Ever	44(91.7)
Never	4(8.3)
Primary tumor location	
Supraglottic cancer	9(18.8)
Glottic cancer	36(75.0)
Infraglottic cancer	3(6.2)
TNM stage	
T-category	
1-2	26(54.2)
3-4	22(45.8)
N-category	
0	36(75.0)
1-3	12(25.0)
M-category	
0	48(100.0)
1	0(0)
Tumor stage	
I-II	23(47.9)
III-IV	25(52.1)
Histological grade	
G1	23(47.9)
G2	16(33.3)
G3	9(18.8)
G4	0(0)
Treatment	
Surgery only	30(62.5)
Surgery + Adjuvant TX^a^	16(33.3)
Others^b^	2(4.2)

^a^ Adjuvant TX: adjuvant radiotherapy and/or chemotherapy.

^b^ Others: palliative treatment and/or treatment at outside institution.

**Table 2:** Association between PRDX3 expression and clinicopathological characteristics in LSCC patients

Variable	*PRDX3 expression*			*P* value
	Grade I	Grade II	Grade III	
Age				
≤65 years	12	9	4	0.929
>65 years	11	7	5	
Sex				
Male	21	15	9	1.000
Female	2	1	0	
Tobacco smoking				
Ever	20	15	9	0.657
Never	3	1	0	
Primary tumor location				
Supraglottic cancer	2	3	4	0.031
Glottic cancer	20	12	3	
Infraglottic cancer	1	1	2	
T-category				
1-2	15	8	3	0.245
3-4	8	8	6	
N-category				
0	21	10	5	0.032
1-3	2	6	4	
Stage				
I-II	16	5	2	0.017
III-IV	7	11	7	
Histological grade				
G1	23	0	0	0.000
G2	0	16	0	
G3	0	0	9	

**Figure 3:**
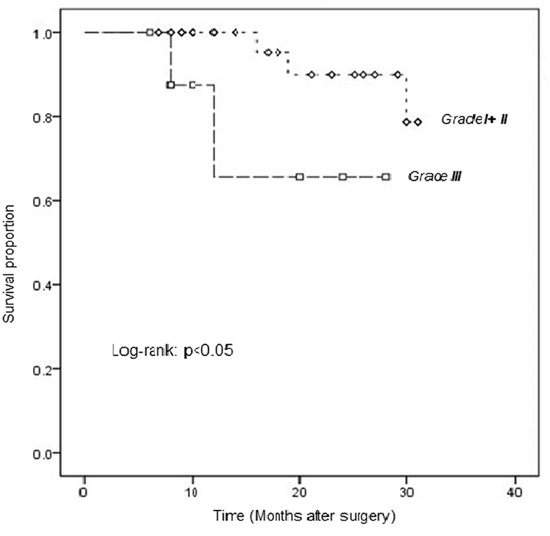
Kaplan-Meier analysis of survival in LSCC patients, stratified according to PRDX3 expression Comparison of overall survival curves for patients with low-moderate (Grade I/II) and high (Grade III) PRDX3 expression in tumors (P=0.042).

Follow-up data for the LSCC patients were available for survival analysis. As shown in Figure 3, the log-rank test revealed a significant difference in survival between the groups with low-moderate (Grade I/II) and high (Grade III) expression of PRDX3 (log-rank, P=0.042).

### Lower expression of PRDX3 associated with inhibition of proliferation and promotion of apoptosis in Hep-2 cells

**Figure 4:**
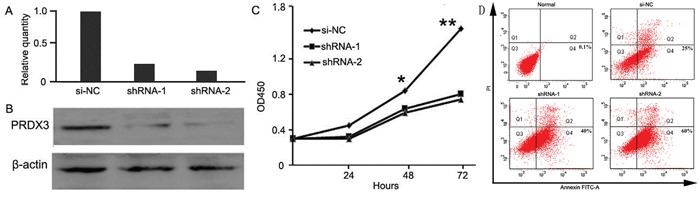
Cell assays of knockdown of PRDX3 in Hep-2 cells Knockdown effect was verified by quantitative RT-PCR **A**. and Western blotting **B. C**. Cell growth was evaluated by Cell Counting Kit-8 (* p<0.05 and **P<0.01). **D**. Cell apoptosis assay was detected by flow cytometry. Normal Hep-2 cells without treatment were used as negative control. Hep-2 cells transfected with si-NC were used as positive control. Apoptosis rates were 0.1%, 25%, 40%, and 60 %, respectively.

Since PRDX3 expression in LSCC was associated with cell differentiation and tumor depth, the possible biological significance of PRDX3 in tumorigenesis was investigated through loss-of-function studies *in vitro*. After thePRDX3 expression level was confirmed at 48 h post-transfection (Figure 4A, 4B), the same number of Hep-2 cells from each group (shRNA-1, shRNA-2, and si-NC group) was inoculated and underwent CCK8 analysis at indicated time points. The result showed knockdown of PRDX3 inhibited the proliferation of Hep-2 cells compared with the si-NC transfectants (Figure 4C). In addition, FITC-Annexin V/propidium iodide staining analysis was performed to determine PRDX3-induced apoptosis, and the percentage of apoptotic cells significantly increased in PRDX3 knockdown cells when compared to control (Figure 4D). These results indicated that inhibition of PRDX3 expression can inhibit cell proliferation and induce cell apoptosis *in vitro*.

### PRDX3 expression in enhancement of migration in Hep-2 cells

**Figure 5:**
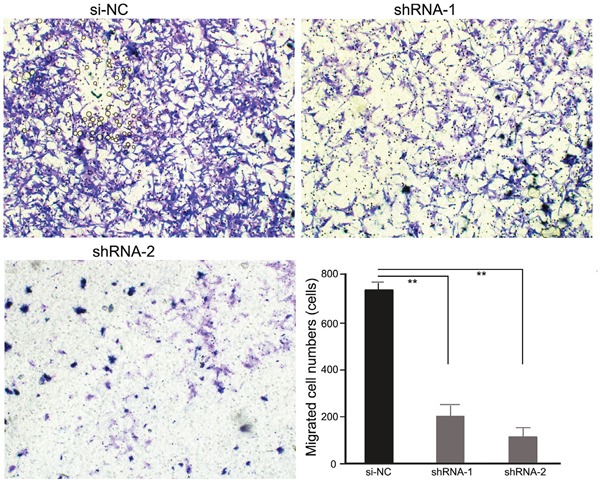
Transwell assays of Hep-2 cells transfected with PRDX3 shRNAs Representative images (magnification, ×100) showed the migration of shRNA-transfected Hep-2 cells. The statistical graph indicates the means ± SEM of the number of cells from 8 random high-power fields (magnification, ×200) counted from three independent experiments.

Migration is an important aspect of tumorigenesis and is closely related to the clinical prognosis. To explore the impact of PRDX3 on LSCC cell migration, a Transwell assay was used to detect the migration ability of Hep-2 cells. As shown in Figure 5, compared with si-NC, Hep-2 cells with knockdown of PRDX3 migrated slower, suggesting that knockdown of PRDX3 inhibited the migration ability of laryngeal cancer Hep-2 cells.

## DISCUSSION

Peroxiredoxins, which reduced intracellular peroxides as a novel kind of antioxidant protein, were extensively expressed in various types of cancers and were thought as a biomarker of cancer cells. The role of PRDXs in LSCC is unknown, although growing evidence suggests their involvement in HNC progression and oxidative stress resistance. Yanagawa et al. found that PRDX I expression was associated with local recurrence and lymph node recurrence of squamous cell carcinoma of the tongue. Their results suggested that PRDX I expression indicates tumors with a high potential for recurrence.[15] Ralhan detected 811 proteins as molecular markers in the screening of head and neck squamous cell carcinoma, including the lower expression of PRDX-2 in head and neck squamous cell carcinoma. The lower expression of PRDX-2 suggests that it may be as a tumor suppressor, of course, its mechanism remains to be further research [16]. However, Zhang performed genotyping analyses for tag SNP of PRDX 1, 2 and 6, and then evaluated the association with susceptibility and clinic stage of esophageal squamous cell carcinoma (ESCC) in a case-control study. Their data suggested that polymorphisms of PRDX 1, 2 and 6 were not associated with esophageal cancer [17]. Our previous study implicated antioxidant proteins in laryngeal carcinoma [7]; thus, the present study investigated the expression profiles and functions of the antioxidant protein family PRDX in laryngeal carcinoma.

All PRDX proteins were found to be expressed in LSCC tumors and adjacent normal tissues, but PRDX3 was significantly up-regulated in tumor tissues, indicating that it may serve as a promising tumorigenesis-associated protein. Therefore, its roles in laryngeal tumorigenesis deserve to be further studied. The immunohistochemistry results were consistent with those of Western blotting and showed PRDX3 was mainly expressed in cytoplasm. The expression of PRDX3 was converse to the degree of histological differentiation: strong immunostaining was observed in poorly differentiated LSCC samples and weak immunostaining in well-differentiated samples.

Most of the patients in our study had early laryngeal cancer, and most of the early laryngeal carcinoma tissue differentiation degree was better. So samples with Grade I expression in our study were the maximum and Grade III were the minimum. We found a significant difference in PRDX3 expression according to N category, tumor stage, and histological grade (p<0.05). We also found a difference of PRDX3 expression in primary tumor location. Just because the glottic cancer was the most common in all laryngeal carcinoma, and moderate, high differentiation were the majority in it. In all cases, weak PRDX3 expression was associated with less-advanced disease and strong expression was associated with more-advanced disease.

The survival curves showed that the prognosis of patients with PRDX3 expression of Grade I+II was better than those of Grade III (p<0.05). It showed that the patients with higher differentiation degree had better prognosis. Maybe we need more cases and longer follow-up to confirm the results further.

PRDX3 was mainly located in mitochondria. Because of the active and indefinite growth of cancer cells and relatively low supply of oxygen, ROS production from mitochondria is accordingly increased [18]. Increased mitochondrial ROS generation and the disturbance of antioxidant systems in cancer cells may lead to oxidative stress and a hypoxic microenvironment, subsequently leading to the induction of apoptosis [19]. Since mitochondrion is the main apoptotic mediator, it is imaginable that control of the ROS level by PRDX3 is involved in apoptotic inhibition. In fact, accumulating studies have suggested that PRDX3 plays an active inhibitory role in chemical-induced oxidation and subsequent apoptosis [19–21]. In the present study we hypothesized that PRDX3 may also be involved in laryngeal tumorigenesis through affecting cell proliferation or apoptosis. We selected the laryngeal tumor cell line Hep-2 to perform *in vitro* functional analyses by constructing knockdown vectors of PRDX3. CCK8 assay showed that knockdown of PRDX3 significantly inhibited the growth of Hep-2 cells. Meanwhile, the down-regulation of PRDX3 could significantly promote cell apoptosis. Migration is an important aspect of tumorigenesis and is closely related to cell proliferation. Transwell assays indicated that knockdown of PRDX3 significantly inhibits Hep-2 cell migration, which may be due to an increase in apoptosis owing to downregulation of PRDX3. The combined results indicated that PRDX3 is a key protein involved in laryngeal tumorigenesis. Since most chemotherapy or radiotherapy for cancer works through an ROS increase and apoptotic induction, our results provide a clue that PRDX3 may be involved in therapy resistance in LSCC.

In conclusion, the present study identified the protein profiles of the PRDX family in LSCC and showed that down-regulation of PRDX3 in Hep-2 cells induced cell apoptosis and inhibited cell proliferation and cell migration. The detailed roles and mechanisms of down-regulated expression of PRDX3 in LSCC warrant further study, and the present study indicates that PRDX3 may be a potential molecular target for novel targeted therapy against LSCC. We will collect more clinical cases and extend the follow-up period to validate PRDX3's roles in clinical diagnosis or assessment of prognosis.

## MATERIALS AND METHODS

### Tissue samples

Tissue samples of LSCC tumors and adjacent healthy tissue were collected from 48 patients who were treated at Yuhuangding Hospital of Qingdao University, Department of Otorhinolaryngology Head and Neck Surgery from 2014 to 2016. The clinical features of these patients are summarized in Table 1. The patients were newly diagnosed with LSCC and had not received any treatment prior to biopsy. The normal tissues adjacent to the tumors were collected at sites more than 2 cm from the edge of the tumor mass and used as a control group. The study protocol was approved by the Committee of Ethics in Research of Yuhuangding Hospital of Qingdao University. All samples were obtained after informed consent was received from the patients. Tumor stage and primary tumor location were defined according to the seventh edition of laryngeal cancer staging international standards revised by the Union for International Cancer Control in 2009 [22]. The primary therapy was surgery in 30 cases, surgery combined with adjuvant radiotherapy and/or chemotherapy in 16 cases, and palliative care and/or treatment at an outside institution in 2 cases. All 48 patients underwent follow-up ranging from 6 to 32 months (mean: 18.9 months).

### Cell line

The LSCC cell line Hep-2 was purchased from the Cell Bank of the Chinese Academy of Medical Sciences (Shanghai, China). Cells were cultured in 6-well plates with a density of 1×10^6^ cells/ml. Cells were cultured with high-glucose DMEM medium (Sigma-Aldrich, St. Louis, MO, USA) supplemented with 10% fetal bovine serum (Gibco BRL, Waltham, MA, USA) in 5% CO_2_ in a humidified atmosphere at 37°C.

### Protein extraction

For protein extraction, tissue samples were frozen in liquid nitrogen and ground into powder. The powder was then collected and dissolved in lysis buffer. Cultured cells were washed twice with cold phosphate-buffered saline, collected, and dissolved in lysis buffer. After sonicated for 2 min separately, samples were allowed to rest at 4°C for 2 h. Centrifugation at 12 000 × g for 45 min at 4°C was then performed. After centrifugation, the supernatant was collected and its protein concentration was measured. Each protein sample was then stored at -80°C until use.

### Western blotting

Western blotting was performed as described previously [23]. Proteins were separated by 12% gel SDS-PAGE, transferred to polyvinylidene difluoride membranes, blocked with 5% (w/v) skim milk for 1 h, and co-incubated for 1 h with the primary antibody of PRDX1 (ab41906, Abcam, Cambridge, UK), PRDX2 (sc-23967, Santa Cruz Biotechnology, USA), PRDX3 (YT3873, ImmunoWay, Plano, TX, USA), PRDX4 (ab59542, Abcam, Cambridge, UK), PRDX5 (ab140926, Abcam, Cambridge, UK), or PRDX6 (sc-134478, Santa Cruz Biotechnology, USA) at room temperature with gentle agitation. The membranes were washed with 0.5% (v/v) Tween-20 in Tris-buffered saline (TBS) 3 times and then incubated with horseradish peroxidase-conjugated anti-IgG for 1 h at room temperature. The immune-reactive complexes were detected using an enhanced chemiluminescence (ECL) kit (Amersham Life Sciences, Marlborough, MA, USA). β-actin (sc-81178, Santa Cruz Biotechnology, USA) was used as an internal standard. The images were analyzed by commercial image analysis software (Gene Tools, version 4.02; Syngeneic, Cambridge, UK). The integrated optical density (IOD) of positive immunostaining was calculated, and the IOD ratio of target protein to β-actin was used to express the results of the Western blot analysis.

### Immunohistochemical staining

Paraffin-embedded tumor tissues and normal adjacent tissues were obtained from Yuhuangding Hospital of Qingdao University. Four-micrometer-thick sections were de-waxed, and then put in 0.01 M citrate buffer (PH=6.0) for antigen retrieval by heating in a microwave oven for 15 min. Endogenous peroxidases of sections were inhibited by incubation with 3% (v/v) H_2_O_2_ for 10 min. Then 3% (w/v) bovine serum albumin in TBS was used to block non-specific binding with antibodies at room temperature for 1 h. Sections were then incubated with PRDX3 overnight at 4°C. After the sections were washed with TBS several times, they were incubated with horseradish peroxidase conjugated anti-rabbit IgG (Zhong-Shan Biotechnology, Zhongshan, Guangdong, China) at a final dilution of 1:200 for 0.5 h at 37°C. Diaminobenzidine (DAB) kit (Zhong-Shan Biotechnology) was used to visualize the peroxidase activity at binding sites. Hematoxylin was used to counterstain the sections. Then the sections were dehydrated and mounted for bright-field microscopy (DM LB2, Leica, Wetzlar, Germany). Pre-immune rabbit IgG was used as a negative control. The intensity of the staining was classified into 3 groups as follows: Grade I, weak staining; Grade II, moderate staining; and Grade III, strong staining.

### Plasmids, shRNAs, and transfection

Two PRDX3 shRNAs (shRNA-1 and shRNA-2) and negative control shRNA (si-NC) were purchased from Shanghai Sangon Biotechnology Company, Shanghai, China. The sequences of shRNA targeting the PRDX3 transcript were as follows: shRNA-1 5’- GATCCTGGAGTCATCAAGCATTTGAGCGTCTTCAAGAGAGACGCTCAAATGCTTGATGACTCCATTTTTTA -3’; shRNA-2 5’-GATCCTTATTCAGCACCAGTTCCTCATGCCTTCAAGAGAGGCATGAGGAACTGGTGCTGAATAATTTTTTA -3’. The PRDX3 gene was amplified using polymerase chain reaction (PCR) with the primers, each containing EcoR I and Xho I sites. The PCR products were digested with EcoR I and Xho I and inserted into a pEGFP-C3 vector. When Hep-2 cells that were seeded onto 6-well plates grew to 70% confluence, the plasmids were transfected into these cells using X-TREME GENE HP DNA Transfection Reagent (Roche, Mannheim, Germany). At indicated time points after the transfection, cells were harvested for further analysis.

### Reverse transcription-polymerase chain reaction (RT-PCR)

RNA of Hep-2 cells with transfection of si-NC or 2 PRDX3 shRNAs were extracted with Tri-zol reagent (Invitrogen, Grand Island, NY, USA) following the manufacturer's instructions. RNA (1ug) was reverse transcribed with 2 U Avian Myeloblastosis Virus Reverse Transcriptase (Promega, Madison, WI, USA), as described by the manufacturer. The primer sequences for PRDX3 were: forward, 5’-GTTGTCGCAGTCTCAGTGG-3’; reverse, 5’-GACGCTCAAATGCTTGATG-3’. β -actin (forward, 5’-ACGTTGACATCCGAAAGACC-3’; reverse, 5’-CCACCGATCCACACAGAGTA-3’) was used as the internal control. PCR products were analyzed by Gene Tools (Syngene, Frederick, MD, USA). Results were shown as the relative quantity of target genes per β-actin. Deionized water was used in place of cDNA as a negative control.

### Cell proliferation assay

Hep-2 cells with transfection of si-NC or 2 PRDX3 shRNAs were seeded at 1×10^3^ cells/well in 96-well plates. Cell viability was assayed by using cell counting kit (CCK)-8 (Dojindo, Kumamoto, Japan) according to the manufacturer's protocols. Briefly, CCK8 reagents were added to the wells at indicated time points and incubated at 37°C for 2 h. The absorbance at 450 nm was recorded with a micro-well plate reader (Synergy TM HT, BIO-TEK, Winooski, VT, USA).

### Cell apoptosis assay

The Annexin V-FITC apoptosis detection kit (BD Biosciences, San Jose, CA, USA) was used to analyze cell apoptosis, following the manufacturer's protocols. In brief, Hep-2 cells, which were transfected with si-NC or 2 PRDX3 shRNAs, and normal Hep-2 cells without treatment were collected after dissociation with ethylenediaminetetraacetic acid (EDTA)-free trypsin and then washed with cold PBS. Then, the cells were re-suspended in the binding buffer with the addition of Annexin V-FITC and propidium iodide for 15 min of incubation in the dark. Finally, flow cytometry analysis was performed immediately thereafter on the BD FACS Calibur (BD Biosciences, San Jose, CA, USA).

### Cell migration

An *in vitro* cell migration assay was performed in 24-well Transwell chambers (Costar, Tewksbury, MA, USA) as previously described [24]. Briefly, the upper and lower culture compartments of each well in the Transwell chambers are separated by polycarbonate membranes (8 μm pore size). Hep-2 cells (2×10^5^) transfected in si-NC or 2 PRDX3 shRNAs in 0.1 ml of serum-free DMEM medium were added to the upper compartment, and 0.6 ml of DMEM containing 10% fetal bovine serum was placed into the lower compartment. Cells were incubated for 4 h at 37°C and stained with 0.1% crystal violet. Non-migrating cells retained on the upper side of the membrane were removed by wiping with a cotton swab. Cells that had migrated through the membrane and had reached the underside of the membrane were counted in 4 microscopic fields using a 20×objective microscope (IX51, Olympus, Japan).

### Statistical analysis

Statistical analyses were performed using SPSS 19.0 software (SPSS Inc., Chicago, IL, USA). Differences among the groups were determined by Fisher's exact test. In all patients, overall survival was defined as the time between the dates of treatment (surgery) and the dates of last follow-up or death. Survival data of patients were analyzed using the Kaplan-Meier estimation method, and the survival curves were evaluated with the log-rank test. P < 0.05 was considered to indicate a statistically significant difference.

## Abbreviations

PRDX: peroxiredoxin
LSCC: laryngeal squamous cell carcinoma
HNC: head and neck cancer
NSCLC: non-small cell lung cancer
ROS: reactive oxygen species
H_2_O_2_: hydrogen peroxide
TBS: tris-buffered saline
IOD: integrated optical density
BSA: bovine serum albumin
RT-PCR: reverse transcription-polymerase chain reaction
CCK: cell counting kit
